# Estrogen-Related Receptor γ Agonist DY131 Ameliorates Lipopolysaccharide-Induced Acute Liver Injury

**DOI:** 10.3389/fphar.2021.626166

**Published:** 2021-04-23

**Authors:** Haoyang Ma, Jiaye Liu, Yang Du, Shengnan Zhang, Weidong Cao, Zhanjun Jia, Wei Gong, Aihua Zhang

**Affiliations:** ^1^ Department of Pediatrics, School of Medicine, Southeast University, Nanjing, China; ^2^ Department of Nephrology, Children’s Hospital of Nanjing Medical University, Nanjing, China; ^3^ Nanjing Key Laboratory of Pediatrics, Children’s Hospital of Nanjing Medical University, Nanjing, China; ^4^ Jiangsu Key Laboratory of Pediatrics, Nanjing Medical University, Nanjing, China

**Keywords:** ERRγ, DY131, lipopolysaccharide, acute liver injury, sepsis

## Abstract

Sepsis-associated liver dysfunction remains a challenge in clinical practice with high mortality and limited specific therapies. DY131 is a pharmacological agonist of the orphan receptor estrogen-related receptor (ERR) γ which plays a crucial role in regulating energy generation, oxidative metabolism, cell apoptosis, inflammatory responses, etc. However, its role in acute liver injury is unknown. In this study, we evaluated the effect of DY131 on lipopolysaccharide (LPS)-induced liver injury. Mice were pretreated with DY131 through intraperitoneal injection at a dose of 5 mg/kg/day for 3 days prior to LPS challenge (10 mg/kg). 24 h later, they were anesthetized and sacrificed. Blood and liver tissues were collected for further studies. In a separate experiment, mice were treated with saline (vehicle) or DY131 for 3 days to evaluate the toxicity of DY131. We found that ERRγ was downregulated in the liver tissues from LPS-treated mice. Pretreatment with DY131 ameliorated LPS-induced liver injury as demonstrated by reduced liver enzyme release (ALT, AST, and LDH), improved liver morphological damage, and attenuated oxidative stress, inflammation and apoptosis. Meanwhile, DY131 had no significant side effects on hepatic and renal functions in mice. Finally, transcriptomics analysis revealed that the dysregulated pathways associated with inflammation and metabolism were significantly reversed by DY131 in LPS-treated mice, providing more evidence in favor of the protective effect of DY131 against LPS-induced liver injury. Altogether, these findings highlighted the protective effect of DY131 on LPS-induced hepatotoxicity possibly via suppressing oxidative stress, inflammation, and apoptosis.

## Introduction

Sepsis is a life-threatening medical emergency and the leading cause of mortality worldwide. Sepsis may cause multiple organ failure which affects the liver, kidneys, lungs, heart, brain, etc (Singer et al., 2016). Among these organ injuries, liver failure serves as an independent risk factor for multiple organ dysfunction and high mortality in patients with sepsis (Woźnica et al., 2018). The overproduction of inflammatory mediators, reactive oxygen species (ROS) and hepatocellular apoptosis are all involved in the disease process (Contreras-Zentella and Hernández-Muñoz, 2016; Tsai et al., 2018). Therefore, drugs targeting oxidative stress and inflammation have long been sought in this setting (Utaipan et al., 2018; Yang et al., 2018). However, the exact pathophysiology of sepsis-associated liver injury has not been completely understood and therapeutic approaches remain limited.

Estrogen-related receptor (ERR) γ is an orphan nuclear receptor that belongs to the ERR subfamily of transcription factors regulating mitochondrial biogenesis/energy generation (Pei et al., 2015), oxidative metabolism (Murray et al., 2013; Fan et al., 2018), cell apoptosis (Gong et al., 2016), inflammatory responses (Ghosh et al., 2019; Kim H.-J. et al., 2019), hormonal/electrolyte homeostasis (Alaynick et al., 2010), etc. ERRγ is primarily expressed in tissues with high metabolic demands such as liver, skeletal muscle, heart, and kidneys (Yang et al., 2006). Studies have demonstrated the potential role of ERRγ in disease conditions such as inflammatory disorders (KimH.-J. et al., 2019; Zhao et al., 2019), alcoholic fatty liver disease (Kim D.-K. et al., 2019), heart disease (Kwon et al., 2013), and carcinomas (Audet-Walsh et al., 2017; Madhavan et al., 2015). In the liver, ERRγ is reported to be associated with hepatic gluconeogenesis and bile acid metabolism (Kim et al., 2014; Zhang et al., 2015). The role of ERRγ in sepsis-induced acute liver injury, however, has not yet been reported.

Lipopolysaccharide (LPS), a component of the gram-negative bacterial cell wall, can lead to severe inflammatory response manifested as the systemic inflammatory response syndrome (SIRS) clinically (Raetz and Whitfield, 2002). LPS is implicated in the pathogenesis of hepatic damage in patients with severe bacteremia via triggering the innate immune response followed by the release of inflammatory cytokines (Kitazawa et al., 2009). Thus, LPS-induced septic animal model was widely used for the mechanistic and therapeutic studies of sepsis-associated liver injury (Zhou et al., 2018). In the present study, we examined the effect of ERRγ agonist DY131 (Kang et al., 2018; Kim H.-J. et al., 2019; Poidatz et al., 2015) on LPS-induced acute liver injury.

## Materials and Methods

### Animal Experiments

All animal procedures were approved by the Nanjing Medical University Institutional Animal Care and Use Committee with the registration number: IACUC-1809017. And the animal studies were performed from August 2018 to September 2020. LPS (L2630, LPS from *E. coli* 0111:B4) and DY131 (GSK9089) were purchased from Sigma-Aldrich (St. Louis, MO, United States) and MedChemExpress (Monmouth Junction, NJ, United States), respectively. DY131 was dissolved in dimethyl sulfoxide (DMSO) and further diluted with saline. Adult male C57BL/6 J mice aged 8–9 weeks were randomly divided into three groups: the control group, LPS group, and DY131 + LPS group. 1) Control group (n = 10): mice received an equal amount of saline; 2) LPS group (n = 10): mice were administered with LPS intraperitoneally (i.p.) at a dose of 10 mg/kg; 3) DY131 + LPS group (n = 10): mice were pretreated with DY131 (5 mg/kg/day, i.p., diluted in 200 μL saline) for 3 days prior to LPS challenge. After 24 h, all mice were anesthetized and sacrificed. Blood and liver tissues were harvested and stored at −80°C or fixed in 4% paraformaldehyde (PFA) for further analyses. The survival rate of mice was monitored for up to 48 h to evaluate the efficacy of DY131 (n = 10 per group). In a separate experiment, mice were treated with saline (vehicle) or DY131 for 3 days to evaluate the toxicity of DY131 *in vivo* (n = 5 in each group).

### Reverse Transcription and Quantitative Real-Time PCR

Total RNA was isolated from liver tissue using the Trizol reagent according to the manufacturer’s instructions (Takara, Japan). cDNA was generated from 1 μg total RNA using PrimeScript RT reagent Kit (RR036, Takara, Japan) following the manufacturer’s instructions. Real-time PCR amplification was performed using SYBR Green Master Mix (Vazyme, Nanjing, China) in the LightCycler^®^ 96 Real-time PCR System (Roche Molecular Systems, Inc.). The relative expression levels of mRNA were normalized to the reference genes and assessed using the 2^−ΔΔCt^ method. The sequences of the primers are shown in Table 1.

**TABLE 1:** Primer sequence.

Gene	Primer sequence
ERRγ	5′-AAG​ATC​GAC​ACA​TTG​ATT​CCA​GC-3′
	5′-CAT​GGT​TGA​ACT​GTA​ACT​CCC​AC-3′
β-actin	5′-GAG​ACC​TTC​AAC​ACC​CCA​GC-3′
	5′-ATG​TCA​CGC​ACG​ATT​TCC​C-3′
NLRP3	5′-ACG​AGT​CCT​GGT​GAC​TTT​GT-3′
	5′-TCT​TCA​GCA​GCA​GCC​CTT​TC-3′
TNFα	5′-TCC​CCA​AAG​GGA​TGA​GAA​G-3′
	5′-CAC​TTG​GTG​GTT​TGC​TAC​GA-3′
IL-6	5′-GCT​TAG​GCA​TAA​CGC​ACT-3′
	5′-GGA​AAT​CGT​GGA​AAT​GAG-3′
IL-1β	5′-ACT​GTG​AAA​TGC​CAC​CTT​TTG-3′
	5′-TGT​TGA​TGT​GCT​GCT​GTG​AG-3′
GAPDH	5-GGT​GAA​GGT​CGG​TGT​GAA​CG-3
	5-CTC​GCT​CCT​GGA​AGA​TGG​TG-3
SOD1	5′-TGA​CTT​GGG​CAA​AGG​TGG​AA-3′
	5′-ATC​CCA​ATC​ACT​CCA​CAG​GC-3′
SOD2	5′-TCA​ATA​AGG​AGC​AAG​GTC​GC-3′
	5′-AAG​GTA​GTA​AGC​GTG​CTC​CC-3′
SOD3	5′-TTC​TTG​TTC​TAC​GGC​TTG​CTA​C-3′
	5′-CTC​CAT​CCA​GAT​CTC​CAG​CAC​T-3′
Bax	5′-CCG​GCG​AAT​TGG​AGA​TGA​ACT-3′
	5′-CCA​GCC​CAT​GAT​GGT​TCT​GAT-3′

### Western Blotting

The liver tissues were lysed in RIPA buffer (Beyotime, P0013B) and supplemented with 1×protease inhibitor cocktail (Roche, 04693132001) for 30 min on ice, followed by centrifugation at 13,000 rpm for 15 min at 4°C. The protein concentration was determined using the BCA Protein Assay Kit (Beyotime, P0010). Immunoblotting was performed using primary antibodies against ERRγ (Santa Cruz, sc-66883, 1:1,000), β-actin (Bioss, bs-0061R, 1:2,500), SOD1 (Proteintech, 67480-1-Ig, 1:5,000), SOD2 (Abclonal, A19576, 1:1,000), SOD3 (Abclonal, A6984, 1:1,000), cleaved caspase-3 (Cell Signaling Technology, 9,661, 1:1,000), and Bax (Cell Signaling Technology, 2,772, 1:1,000), followed by the incubation of peroxidase-conjugated goat anti-rabbit secondary antibody (Beyotime, A0208, 1:2,000). The blots were visualized using the Amersham Enhanced Chemiluminescence detection system (Bio-Rad, Hercules, CA, United States).

### Histological Analysis and Immunohistochemistry

Harvested liver tissues were cut into small pieces and fixed in 4% PFA and embedded in paraffin according to the standard procedure for further histological analysis. Thereafter, 4 μm sections were prepared for H&E and periodic acid-Schiff (PAS) staining. The liver histopathological evaluation was performed in a blinded manner as described before (Ishak et al., 1995). For details, inflammation was analyzed in portal, periportal, and acinar regions based on the presence of neutrophil or eosinophil granulocytes. Analyses of focal lytic necrosis, confluent necrosis and hemorrhage were also included. For immunohistochemistry, briefly, the paraffin-embedded liver slides (4 μm) were deparaffinized, rehydrated, blocked with 3% H_2_O_2_ for 20 min at room temperature, and washed with 1× PBS. Antigen retrieval was performed by boiling sections in 1× improved citrate antigen retrieval solution (Beyotime, P0083). After being washed with 1× PBS, the sections were incubated with blocking buffer (Beyotime, P0102) for 1 h at room temperature and then incubated with primary antibodies, including ERRγ (Abclonal, A16373, 1:50), IL-6 (Proteintech, 21865-1-AP, 1:50) and IL-1β (Proteintech, 16806-1-AP, 1:50) in a wet box at 4°C overnight. After being washed with 1× PBS, the sections were incubated with a peroxidase-conjugated Polymer (Dako REAL EnVision/HRP, K5007) at room temperature for 60 min. After being washed with 1× PBS, the staining was visualized using freshly prepared Dako REAL DAB + Chromogen (Dako K5007, Denmark). The nuclei were counterstained with hematoxylin staining solution at room temperature. Following washing and dehydration, the slides were mounted with coverslips, and the staining was evaluated under an Olympus BX51 microscope (Olympus, Center Valley, PA, United States).

### Blood Biochemistry

Serum samples were collected from blood by centrifugation at 4,000 rpm for 20 min and stored at −80°C. Serum levels of alanine aminotransferase (ALT), aspartate aminotransferase (AST), lactate dehydrogenase (LDH), creatinine (Cr) and blood urea nitrogen (BUN) were evaluated using Hitachi 7600 modular chemistry analyzer according to the manufacturer’s instructions (Hitachi Ltd., United States).

### ELISA

Serum levels of inflammatory cytokines were measured using the mouse TNFα ELISA kit (Dakewe Biotech, 1217202) and IL-6 ELISA kit (Dakewe Biotech, 1210602) according to the manufacturer's protocols.

### Determination of Glutathione and Malondialdehyde in Liver Tissues

For GSH measurement, liver tissues were homogenized in the indicated solutions according to the manufacturer’s instructions. The supernatants were obtained by centrifugation at 10,000 g for 10 min at 4°C. A GSH quantification kit (Beyotime, S0052) was used for the determination of GSH content in liver tissues. For MDA measurement, liver tissues (approximately 100 mg) were homogenized in 1 ml PBS containing 1 mM EDTA and centrifuged at 10,000 g for 10 min at 4°C. MDA content was determined by the MDA quantification kit (Jiancheng, A003-1) according to the manufacturer’s instructions. MDA value was normalized to the liver protein level determined by the BCA Protein Assay Kit (Beyotime, P0010).

### Dihydroethidium Staining

To measure *in situ* ROS levels, frozen liver sections (5 μm) were prepared with a Leica CM1900 Cryostat (Leica, Germany) and stained with DHE solution (3 μM) (Beyotime, S0063) for 30 min in the dark at 37°C. Then they were washed three times with 1× PBS. Fluorescence images were captured under 543 nm excitation light with a laser scanning confocal microscope (CarlZeiss LSM710, Germany). The staining density was quantified by ImageJ software (Bethesda, MD).

### Terminal Deoxynucleotidyl Transferase dUTP Nick End Labeling Assay

*In situ* apoptotic cells were detected in paraffin-embedded liver sections using a TUNEL BrightGreen Apoptosis Detection Kit, according to the manufacturer’s protocols (Vazyme, A112-03). Nuclei were stained by 4′6-diamidino-2-phenylindole (DAPI) dye (Beyotime, C1005). Images were obtained using a laser scanning confocal microscope (CarlZeiss LSM710, Germany). The number of TUNEL-positive cells was averaged over at least two randomly selected fields per section.

### Transcriptome Sequencing and Gene Expression Analysis

RNA sequencing (RNA-Seq) and gene expression analysis were performed by BioNovoGene (Suzhou, China). Three randomly selected samples in each group were subjected to RNA-Seq experiment. Briefly, RNA quantification and qualification were assessed before cDNA library construction and sequencing. A total amount of 1 µg RNA per sample was used as input material for the RNA sample preparation. Sequencing libraries were generated using NEBNext Ultra Directional RNA Library Prep Kit for Illumina (NEB, United States) following the manufacturer’s protocol. The quality of the constructed libraries was assessed on the Agilent Bioanalyzer 2100 system (Agilent Technologies, Santa Clara, CA, United States). Qualified libraries were sequenced on an Illumina Novaseq platform and 150 bp paired-end reads were generated. The raw reads were processed under quality control before they were aligned to the reference genome using Hisat2 v2.0.5. The gene expression was measured in units of fragments per kilobase of exon model per million mapped reads (FPKM). The analysis of differentially expressed genes (DEGs) was performed using the DESeq2 R package (1.16.1). The *p* value was adjusted using the Benjamini and Hochberg’s approach for controlling the false discovery rate (FDR). Genes with an adjusted *p* value (*p*_adj_) < 0.05 were assigned as DEGs. All original sequence datasets have been submitted to the database of Gene Expression Omnibus (GEO) with an accession link (https://www.ncbi.nlm.nih.gov/geo/query/acc.cgi?acc=GSE160880).

To further explore the gene expression dynamics and functions as well as the regulatory networks involved, the series test of cluster (STC) algorithms and Kyoto Encyclopedia of Genes and Genomes (KEGG) pathways were performed according to the protocols previously described (Clarke et al., 2008; Gao et al., 2014; Miller et al., 2002). DEGs at a logical sequence according to the random variance model corrective ANOVA were selected and sixteen model pattern profiles were used to summarize. Expression patterns of genes which showed significant *p* values (< 0.05) were identified and two profiles with statistical significance (Numbered 5 and 8, respectively) were chosen for further hierarchical clustering and KEGG analyses.

### Statistical Analysis

Data were expressed as mean ± SEM. For statistical analyses, the unpaired Student’s *t*-test and one-way ANOVA were performed with GraphPad Prism (6.01, San Diego, CA, United States). The level of statistical significance was set to *p* < 0.05.

## Results

### ERRγ Was Downregulated in the Livers From LPS-Treated Mice

**FIGURE 1:**
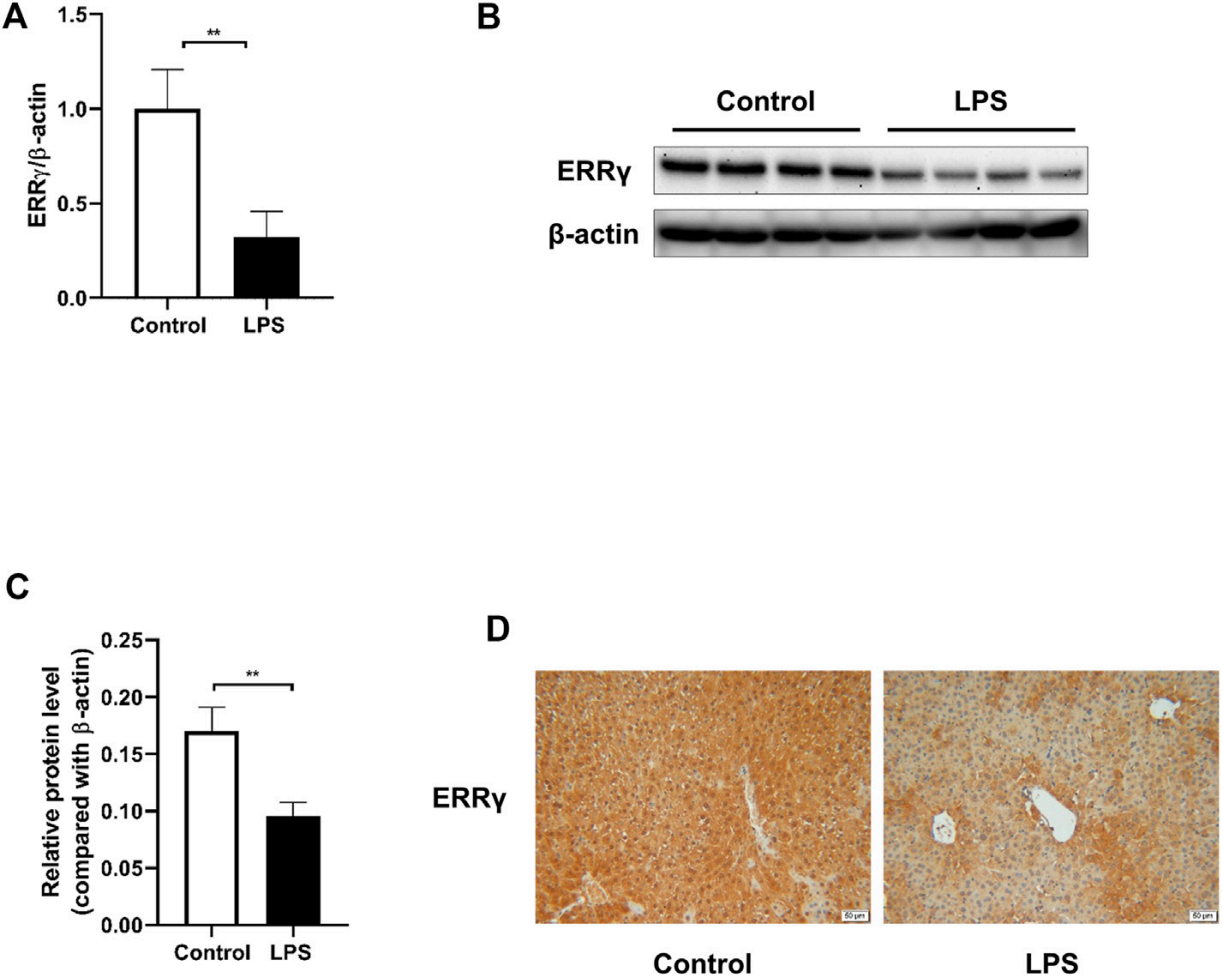
LPS challenge downregulated ERRγ expression in mouse livers. LPS at the dose of 10 mg/kg or saline (control) was administered to the male C57BL/6 J mice intraperitoneally, then mice were sacrificed after 24 h. **(A)** mRNA expression of ERRγ determined by qRT-PCR (n = 10 in each group). **(B)** Western blot of ERRγ (n = 8 in each group). **(C)** Quantitative analysis of ERRγ by densitometry (n = 8 in each group). **(D)** Representative images of immunohistochemical staining of ERRγ in livers from mice in the control and LPS groups (magnification ×200, scale bar: 50 μm, n = 5 in each group). Data were presented as means ± SEM. ^**^ *p* < 0.01 vs. control group.

First, we investigated the expression of ERRγ in liver tissues from LPS-treated mice. Through qRT-PCR and Western blotting (Figures 1A–C), we found a significant decrease of ERRγ in the livers from LPS-treated mice both at mRNA and protein levels. Such a downregulation was further confirmed by immunohistochemistry (Figure 1D). These data suggested that the reduction of ERRγ in the liver might be associated with the septic liver injury.

### Pretreatment With ERRγ Agonist DY131 Attenuated LPS-Induced Liver Injury in Mice

**FIGURE 2:**
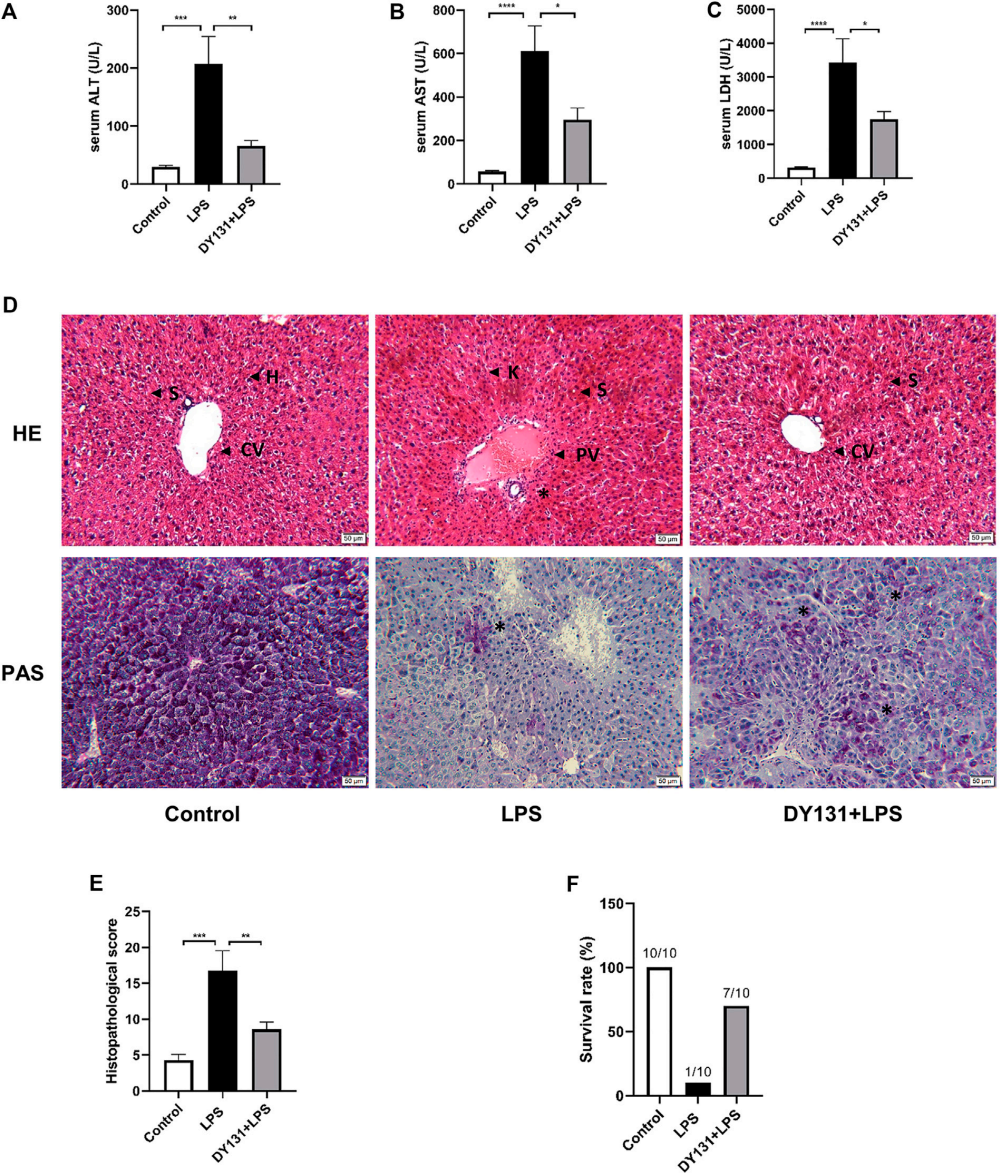
Pretreatment with DY131 attenuated LPS-induced liver injury. Mice were pretreated with DY131 prior to LPS challenge as described in materials and methods. **(A–C)** Serum levels of ALT, AST, and LDH (n = 10 in each group). **(D)** Representative images of H&E and PAS staining of liver sections (magnification ×200, scale bar: 50 μm). CV, central vein; H, hepatocytes; S, sinusoids; PV, portal vein; K, Kupffer cells; asterisks* indicated inflammatory infiltrations in portal areas (H&E) and PAS-positive materials in hepatocytes (purple-stained). **(E)** Analysis of liver histopathological score in different groups based on H&E staining images (n = 4–7 in each group). **(F)** Survival rate at the 48 h after LPS challenge. Data were presented as means ± SEM. ^*^ *p* < 0.05, **p < 0.01, ***p < 0.001, and ****p < 0.0001 vs. the indicated group.

To determine the effect of DY131 on septic liver injury induced by LPS, mice were pretreated with DY131 before LPS administration. As shown by the data, serum levels of ALT, AST, and LDH were significantly elevated in LPS-treated mice compared with those in the control group. Strikingly, DY131 pretreatment significantly decreased the elevation of these liver enzymes (Figures 2A–C). As shown in the H&E staining results, normal hepatic architecture with central vein and surrounding hepatocytes were displayed in the liver of control mice, while the liver from LPS-treated mice showed congestion of central vein, blood sinusoids and increased infiltration of inflammatory cells in the portal areas. However, all these morphological changes were attenuated in the livers from DY131-treated mice (Figure 2D). Besides, PAS staining was used to examine the liver glycogen of mice. As shown in Figure 2D, strong PAS-staining was shown in the liver from control mice, which indicated the abundant glycogen in liver. However, the level of hepatic glycogen was dramatically reduced in LPS-challenged mice, as demonstrated by the scattered and sporadic purple-stained cells in liver. Interestingly, DY131 treatment could partially restore the glycogen in liver, indicating a potential protective effect of DY131 on hepatic gluconeogenesis or storage. Furthermore, the liver histopathological scores (based on H&E staining) using the modified histological activity index (HAI) (Ishak et al., 1995) were estimated, showing a lower injury score in mice with DY131 pretreatment as compared with those in the LPS group (Figure 2E). The survival rate at the 48 h after LPS challenge was estimated. As shown in Figure 2F, 9 of 10 mice died at 48 h after LPS treatment, while in DY131-treated group, only 3 of 10 mice died at the same time. This result indicated that DY131 treatment increased the survival rate of LPS-treated mice. Taken together, these results indicated that pretreatment with DY131 could ameliorate LPS-induced liver injury in mice.

### DY131 Pretreatment Alleviated Oxidative Stress in LPS-Treated Mice

**FIGURE 3:**
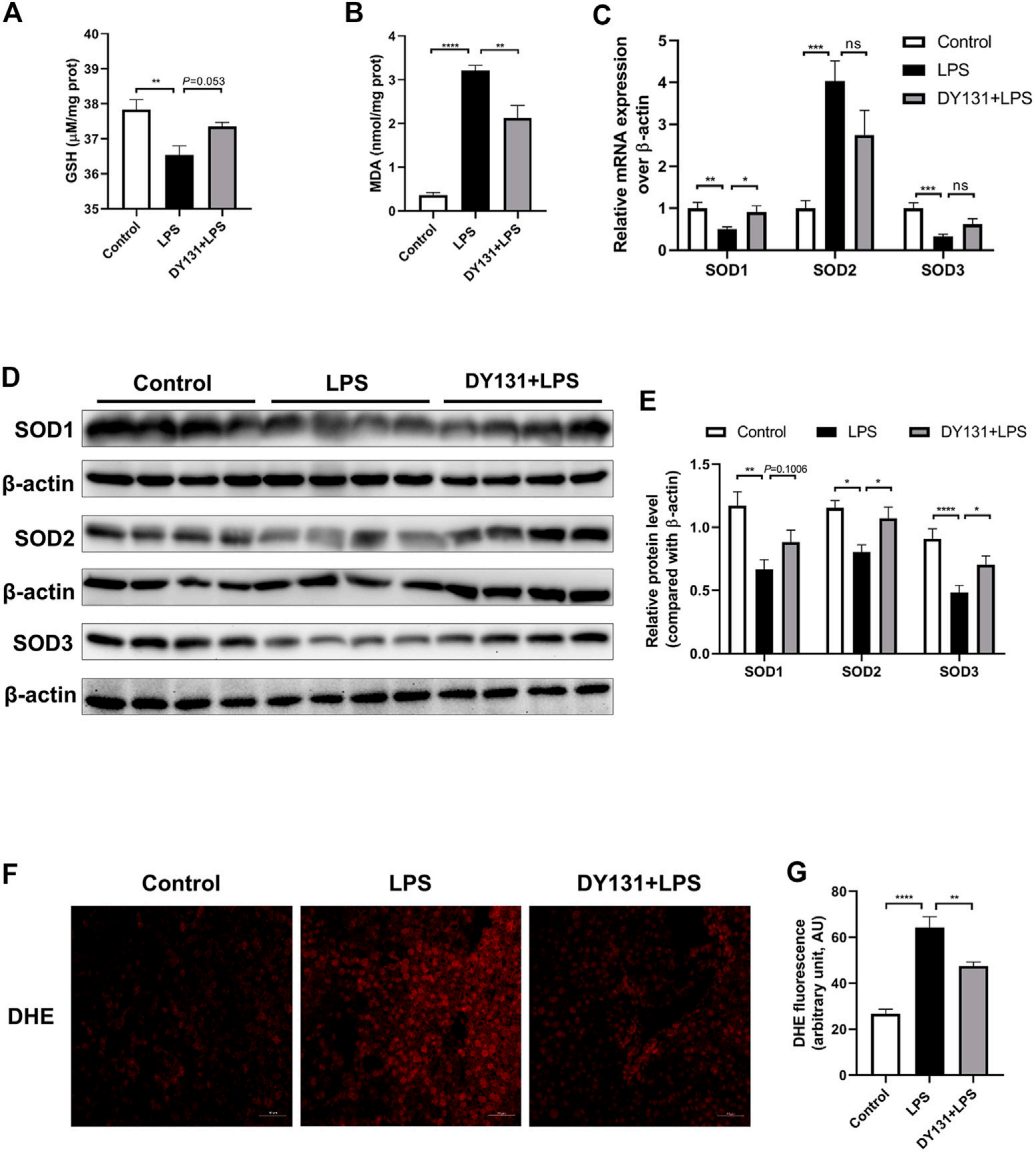
DY131 ameliorated oxidative stress in LPS-treated mice. **(A,B)** The levels of the antioxidant GSH and oxidative stress marker MDA in livers were measured using a commercial kit (n = 10 in each group). **(C)** mRNA expressions of SOD1, SOD2, and SOD3 were determined by qRT-PCR (n = 10 in each group). **(D)** Protein levels of SOD1, SOD2, and SOD3 were detected by Western blotting (n = 8 in each group). **(E)** Quantitative analyses of SOD1, SOD2, and SOD3 by densitometry (n = 8 in each group). **(F)** Representative images of DHE staining of liver tissues in different groups (magnification ×200, scale bar: 50 μm, n = 3 in each group). **(G)** Quantification of the mean fluorescence intensity of DHE was analyzed by ImageJ software. Data were presented as means ± SEM. ^*^ *p* < 0.05, **p < 0.01, ***p < 0.001 and ****p < 0.0001 vs. the indicated group. NS, no significance.

Accumulating evidence has suggested that ERRγ plays a critical role in the regulation of oxidative metabolism and that loss of ERRγ caused metabolic defects and oxidative stress (Alaynick et al., 2007; Fan et al., 2018; Murray et al., 2013). Here we examined the potential effect of DY131 on the oxidative stress in the livers of LPS-treated mice. As expected, LPS challenge depleted hepatocytic GSH and enhanced the oxidative stress marker MDA, while DY131 pretreatment could partially restore the level of GSH and decrease the level of MDA (Figures 3A,B). Meanwhile, LPS administration resulted in a remarkable reduction of SOD1 and SOD3 at mRNA levels, which was partially reversed by DY131 (Figure 3C). Western blotting further revealed that DY131 pretreatment restored the reduced SODs induced by LPS to some degree (Figures 3D,E). DHE staining was also performed to assess hepatocytic ROS levels in different groups. As shown in Figures 3F,G, the enhanced production of ROS induced by LPS in the liver was attenuated by DY131. These results suggested that ERRγ agonist DY131 could improve liver function through inhibiting LPS-induced oxidative stress.

### DY131 Pretreatment Ameliorated the Inflammatory Response in LPS-Treated Mice

**FIGURE 4:**
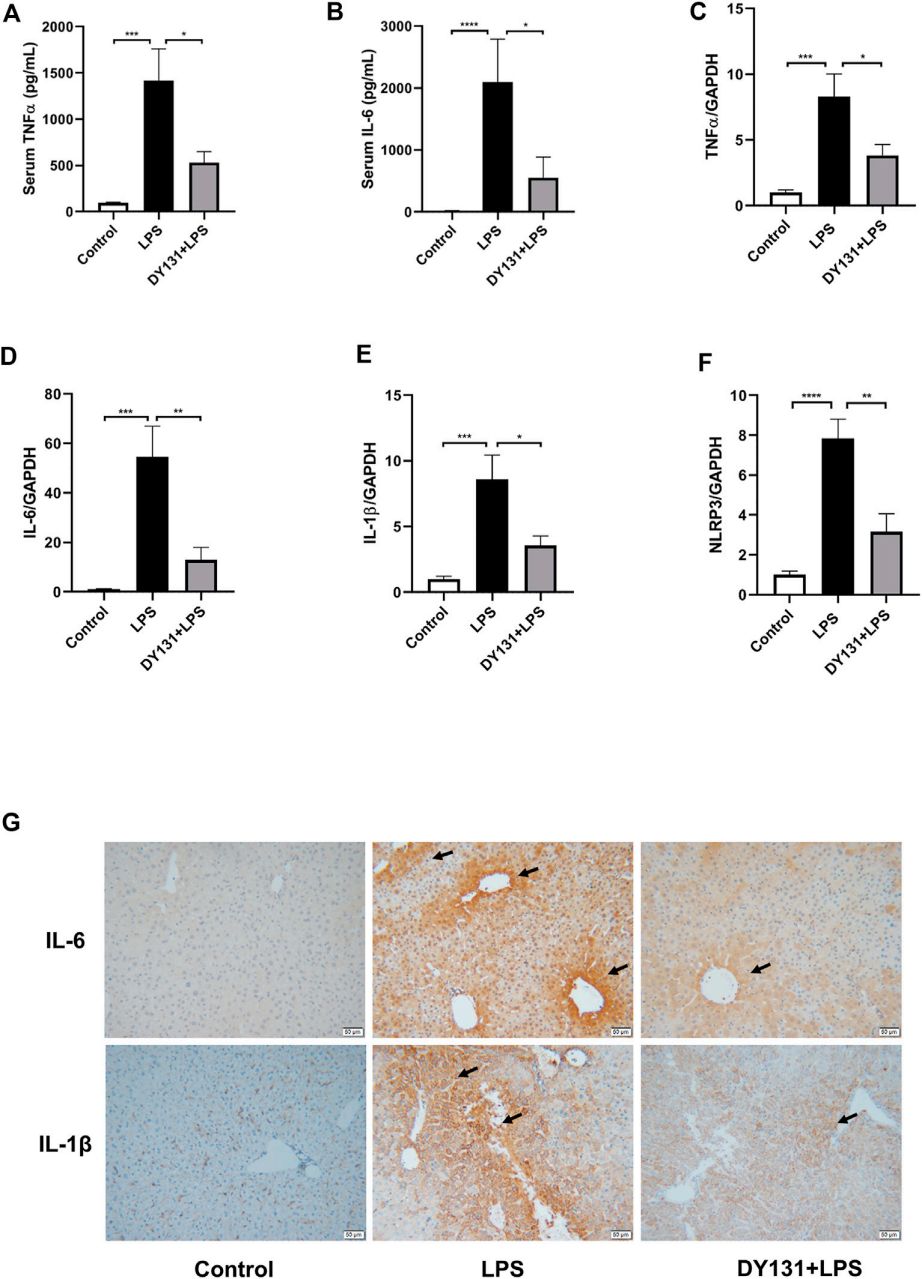
DY131 alleviated the inflammatory response in LPS-treated mice**. (A,B)** Serum levels of TNFα and IL-6 measured by ELISA kits (n = 10 in each group). **(C–F)** mRNA expressions of TNFα, IL-6, IL-1β and NLRP3 determined by qRT-PCR (n = 10 in each group). **(G)** Representative images of immunohistochemical staining of IL-6 and IL-1β in liver tissues (magnification ×200, scale bar: 50 μm, n = 4-5 in each group). Data were presented as means ± SEM. ^*^ *p* < 0.05, **p < 0.01, ***p < 0.001, and ****p < 0.0001 vs. the indicated group.

The development of LPS-induced hepatocellular injury is closely linked with the enhanced inflammation in the liver and circulation (He and Karin, 2011). In the present study, serum levels of TNF-α and IL-6 were significantly increased in the LPS group, while pretreatment with DY131 effectively suppressed the levels of these cytokines (Figures 4A,B). Consistently, the mRNA expressions of TNF-α, IL-6 and IL-1β were all markedly increased in the livers of mice challenged with LPS, which was significantly blunted by DY131 pretreatment (Figures 4C–E). We also detected the expression of NLRP3, the key component of NLRP3 inflammasome. As shown in Figure 4F, the expression of NLRP3 induced by LPS in liver tissue was decreased by DY131 treatment. Furthermore, as demonstrated by immunohistochemistry, the hepatic expressions of IL-6 and IL-1β were negligible in the liver from control mice, whereas strong expressions of both the two cytokines were visible surrounding the central vein and portal area in LPS-treated mice. In DY131-treated mice, however, reduced positive-staining of hepatic IL-6 and IL-1β was respectively observed in liver. (Figure 4G). These data indicated a potent effect of DY131 against the inflammation caused by LPS in the liver and circulation.

### DY131 Protected Against the Apoptosis of Hepatocytes in LPS-Treated Mice

**FIGURE 5:**
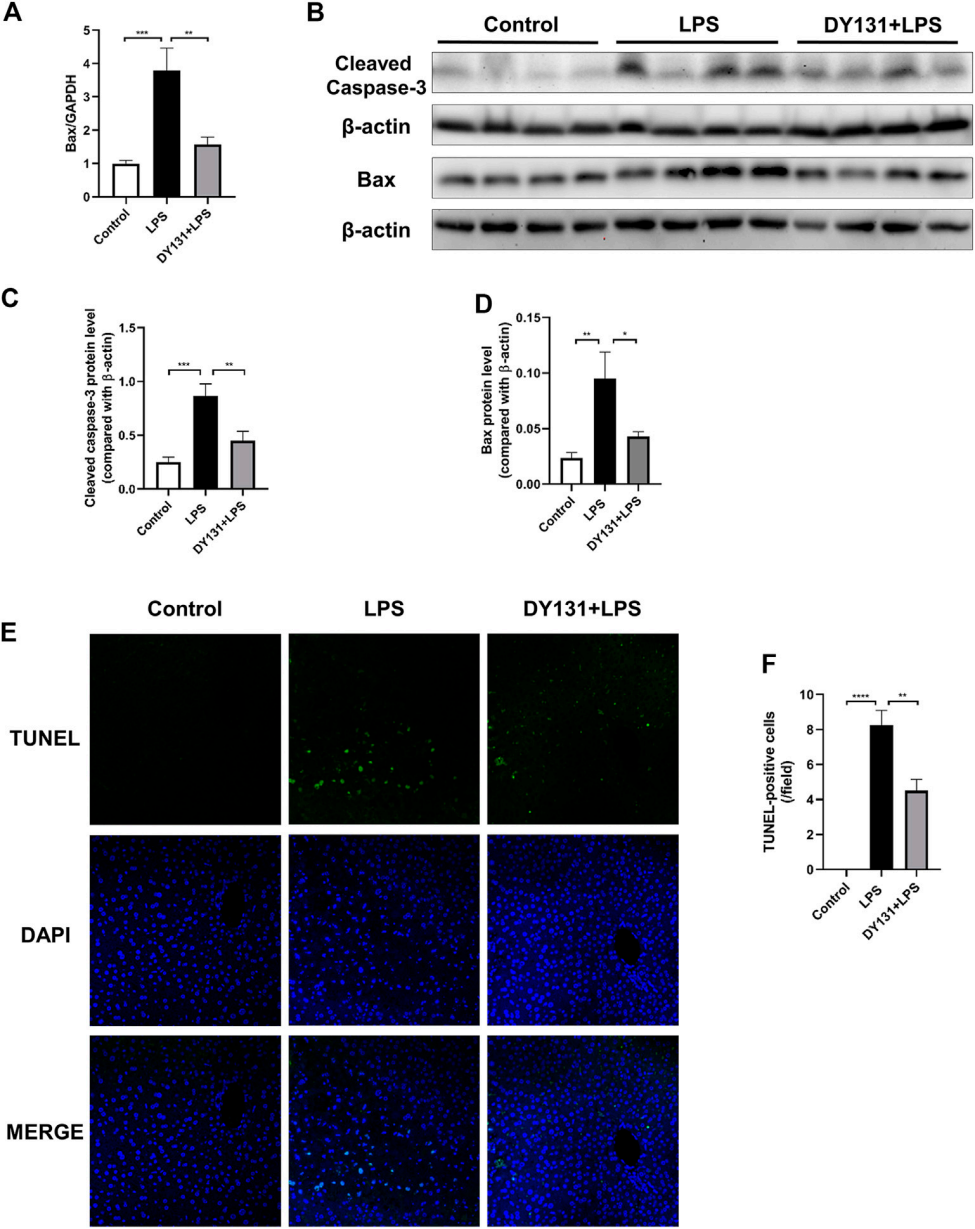
DY131 attenuated LPS-induced hepatic apoptosis. **(A)** mRNA expression of Bax determined by qRT-PCR (n = 10 in each group). **(B)** Protein levels of cleaved caspase-3 and Bax detected by Western blotting (n = 8 in each group). **(C,D)** Quantitative analyses of cleaved caspase-3 and Bax by densitometry (n = 8 in each group). **(E)** Representative images of TUNEL staining of liver tissue sections. Green staining indicated apoptotic cells and DAPI staining was used to visualize nuclei (magnification ×200, scale bar: 50 μm, n = 4 in each group). **(F)** Number of TUNEL-positive cells per field in different groups. Data were presented as means ± SEM. *p < 0.05, **p < 0.01, ***p < 0.001 and *****p < 0.0001 vs. the indicated group.

Hepatocytic apoptosis occurred at early reversible and irreversible stages in response to various stimuli including LPS challenge (Najimi et al., 2009; Zhou et al., 2018). In the present study, the upregulated mRNA expression of Bax in the livers of LPS-treated mice was significantly decreased by DY131 pretreatment (Figure 5A). Consistently, the enhanced protein levels of cleaved caspase-3 and Bax induced by LPS were suppressed by DY131 (Figures 5B–D). Moreover, the increased number of TUNEL-positive hepatocytes in LPS-treated mice were obviously lowered by DY131 pretreatment (Figures 5E,F). These data suggested that ERRγ agonist DY131 could protect against hepatocytic apoptosis in LPS-treated mice.

### Evaluation of DY131 Toxicity in Mice

**FIGURE 6:**
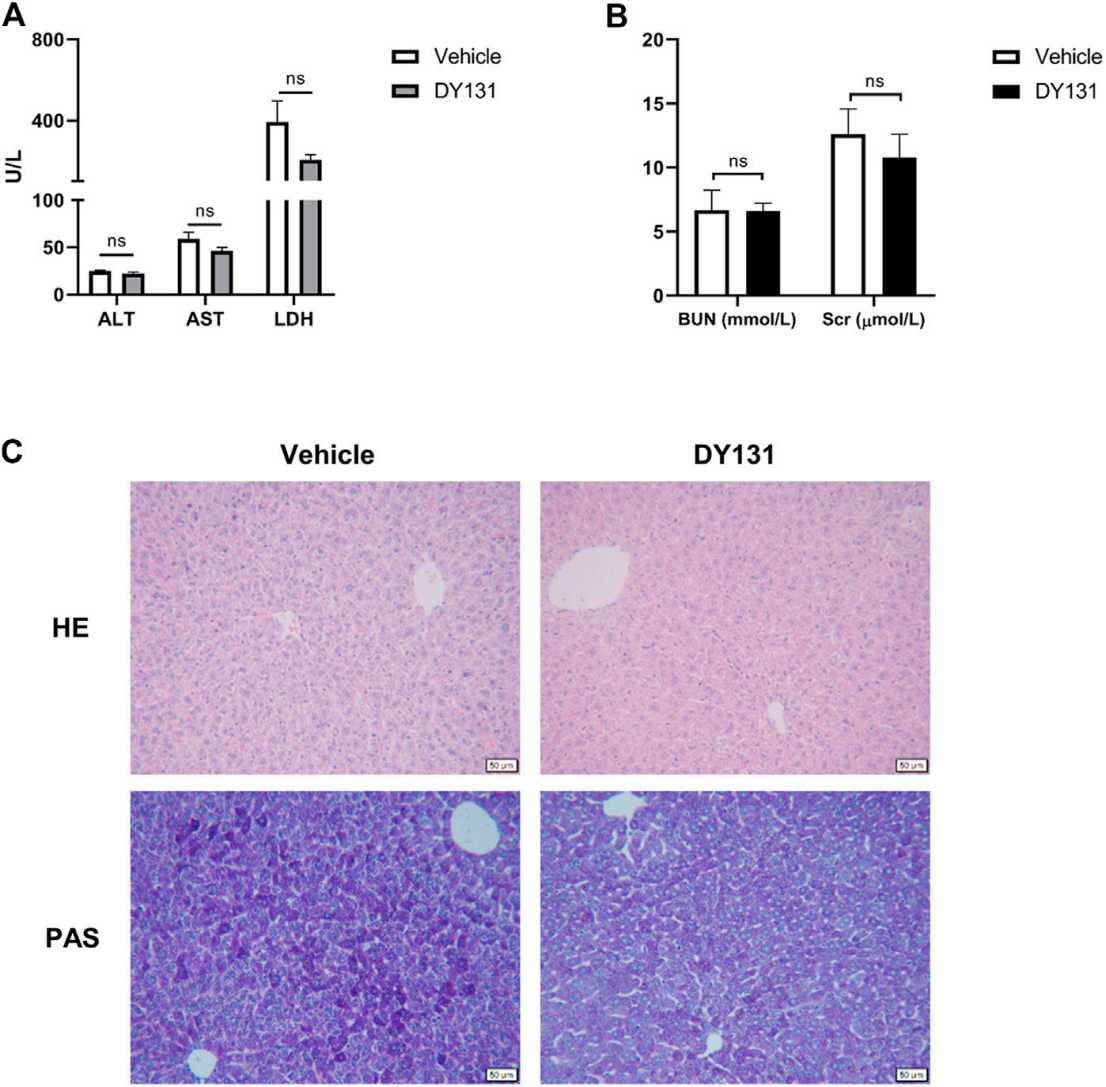
Evaluation of the in vivo toxicity of DY131 at the dose of 5 mg/kg. Adult male C57BL/6 J mice were intraperitoneally administered with DY131 (5 mg/kg/day, dissolved in 200 μL saline) or 200 μL saline (vehicle) for three days. **(A,B)** Serum levels of ALT, AST, LDH, Cr and BUN (n = 5 in each group). **(C)** Liver sections were stained with H&E and PAS (magnification ×200, scale bar: 50 μm). Data were presented as means ± SEM. NS, no significance.

In the present study, we applied a relatively lower dose of DY131 (5 mg/kg) in the mice. To determine the potential drug adverse effects, liver and renal functions were analyzed from vehicle (saline) group and DY131 treatment group, respectively. As shown by the data, DY131 had no obvious side effects with regards to the hepatic and renal functions (Figures 6A,B). Furthermore, H&E and PAS staining of liver tissues showed no difference between both groups in morphology and glycogen levels, respectively (Figure 6C). These data indicated that DY131 at the current dosage had no obvious toxicity in mice.

### DY131 Pretreatment Reversed the Altered Inflammatory and Metabolic Pathways in Livers of LPS-Treated Mice

**FIGURE 7:**
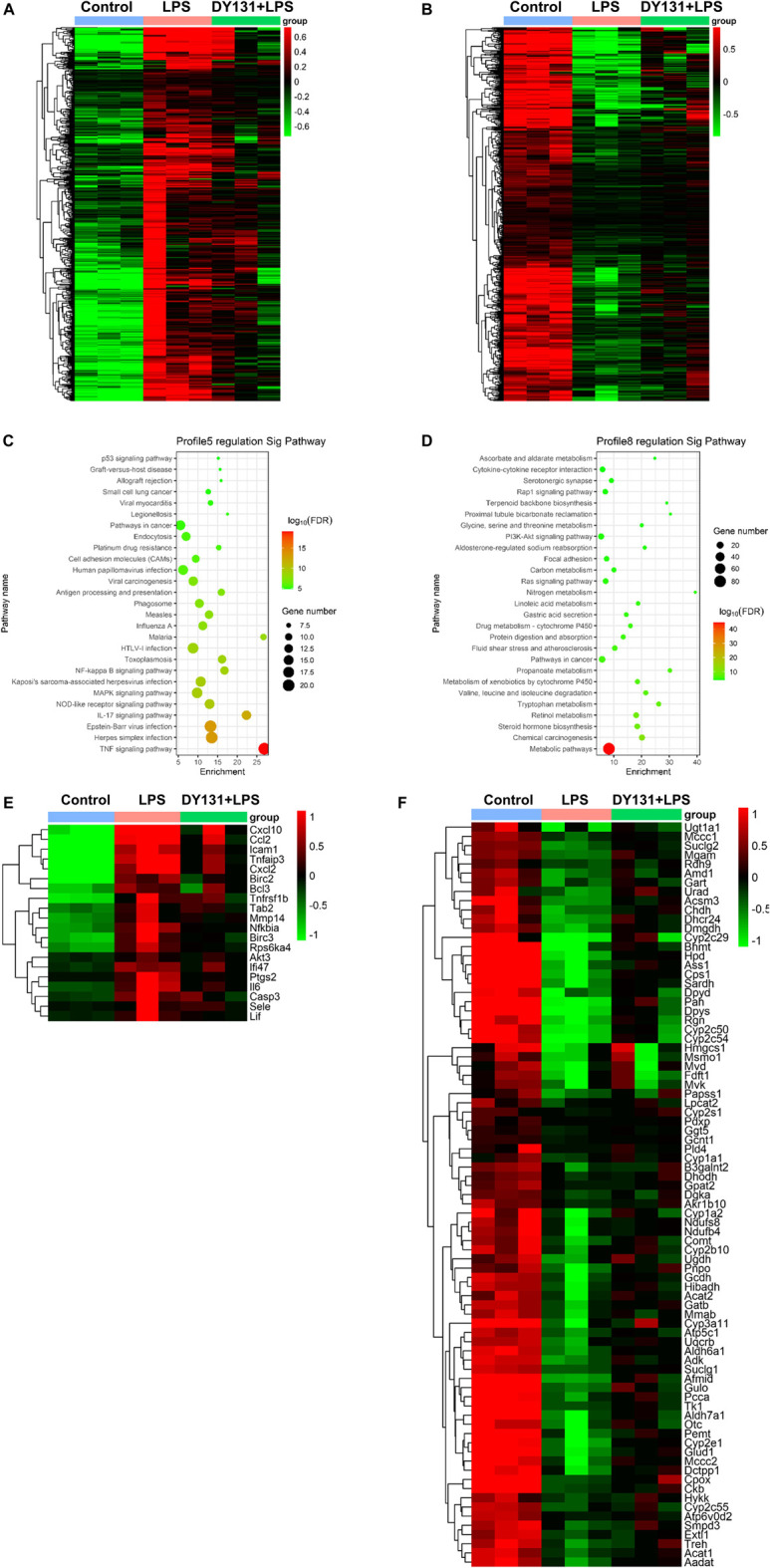
Transcriptomics analysis. **(A,B)** Hierarchical clustering analyses of DEGs in Profile 5 **(A)** and Profile 8 **(B)**. **(C,D)** The KEGG pathway analysis of DEGs in Profile 5 and Profile 8. Red and green dots indicate upregulated and downregulated transcripts, respectively. **(E,F)** Hierarchical clustering analyses of the top one significantly changed pathway in Profile 5 (TNF signaling pathway) and Profile 8 (metabolic pathway).

**FIGURE 8:**
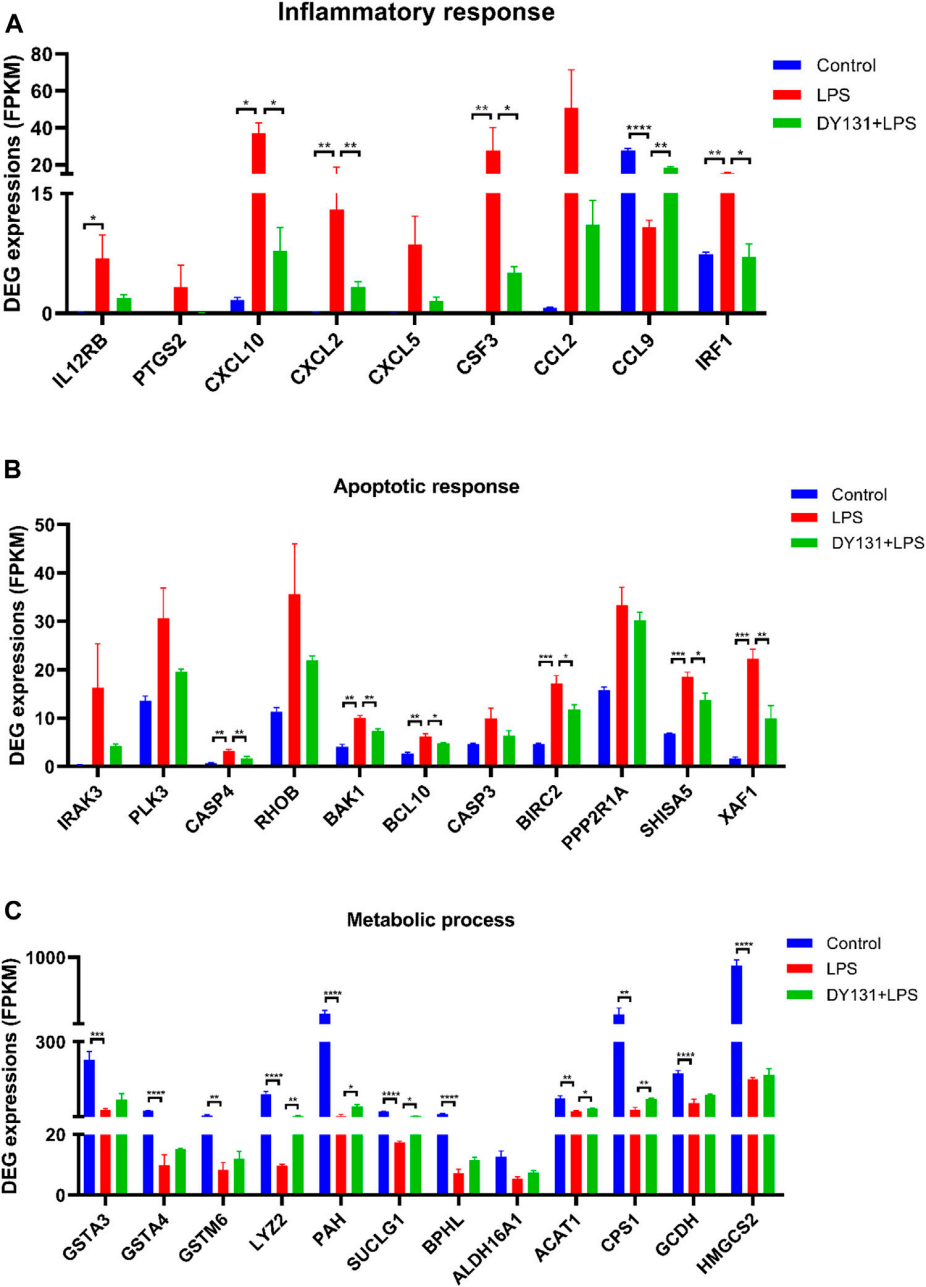
Expression patterns of genes involved in the inflammation, apoptosis and metabolism. **(A)** Genes related to inflammatory response. **(B)** Genes involved in apoptosis response. **(C)** Genes associated with metabolic process. Data were presented as means ± SEM according to the RNA-Seq results (FPKM value). *p < 0.05, **p < 0.01, ***p < 0.001 and ****p < 0.0001 vs. the indicated group (n = 3 per group).

To better investigate the effect of DY131 in LPS-induced septic model, an RNA-seq analysis was performed to detect the DEGs in the livers from different groups. In total, 4,918 DEGs were found between control and LPS groups, and 143 DEGs were found between LPS and DY131 + LPS groups [Fold Change >2, *p* value < 0.05 (Supplementary Figure S1A)]. The Venn diagram (Supplementary Figure S1A) indicated that 119 DEGs were significantly altered in all three groups. To further examine the expression change patterns of the 119 DEGs, STC algorithms of gene expression dynamics were used and the 119 genes were placed into 16 expression pattern profiles (Supplementary Figure S1B). A total of five expression patterns including Profiles 3, 5, 7, 8, and 13 were statistically significant (Supplementary Figure S1B). Genes in Profile 5 were gradually upregulated in the LPS group and then downregulated in the DY131-treated group, while Profile 8 was comprised of genes that are suppressed in the LPS group and reversed in the DY131-treated group. Genes contained in these two profiles were displayed respectively in the hierarchical clustered heat maps which demonstrated the general variations between LPS and DY131 + LPS groups (Figures 7A,B). These data indicated that DY131 could reverse the LPS-induced gene expressions at transcriptional level. Furthermore, KEGG analysis revealed various enrichment-related pathways according to the genes derived from Profile 5 and Profile 8 (Figures 7C,D). The top one pathways significantly enriched in Profiles 5 and Profile 8 were the TNF signaling pathway and metabolic pathway, respectively, suggesting that the inflammatory events were inhibited while the metabolism was improved by DY131 treatment (Figures 7C–F). Besides, we further analyzed the expressions of some inflammatory, apoptotic and metabolic genes determined by RNA-seq (FPKM value) and observed the consistent trends (Figures 8A–C). All these data provided a comprehensive evidence that DY131 could alleviate inflammation and apoptosis, as well as improve metabolic status in the livers of mice subjected to LPS.

## Discussion

The liver acts as a critical organ in the defense system against circulating microbial pathogens through orchestrating a variety of cells to combat infection (Jenne and Kubes, 2013). Physiologically, homeostatic inflammatory processes take place and are tightly regulated in a coordinated way, which is essential to maintain tissue and organ homeostasis (Protzer et al., 2012; Robinson et al., 2016). However, when exposed to systemic and excessive inflammation, the liver plays a central role in the initiation of sepsis by magnifying oxidative stress and inflammatory cytokines (Cichoż-Lach and Michalak, 2014; Larrouyet-Sarto et al., 2020). On the other hand, studies also suggested that the alterations of hepatic metabolism contributed to sepsis-related liver injury (Feng et al., 2013; Geng et al., 2020). The altered metabolic pathways identified in the liver during sepsis included arginine/proline/glutathione/glycine/purine metabolisms, bile acid biosynthesis and so on. All these metabolic changes could result in cholestasis and hepatocellular injury (Ilaiwy et al., 2019).

ERRγ could be induced by many factors including some metabolic responses. For example, HIF-1α could regulate ERRγ expression to respond to the hypoxia in liver (Lee et al., 2012). ERRγ also can be induced when there are metabolic demands, such as the physical exercise in the skeletal muscle (Rangwala et al., 2010). On the other hand, recent evidence indicated a pivotal role of ERRγ as a regulator of mitochondrial biogenesis, oxidative phosphorylation, fatty acid oxidation, and especially the metabolic pathways (Alaynick et al., 2007; Giguère, 2008; Islam et al., 2020). Although ERRγ is abundantly expressed in the liver, its exact role in liver remains elusive (Misra et al., 2017). Considering the multiple role of ERRγ in the cellular stresses and the metabolic pathways which are involved in the liver damage, we suppose that ERRγ might be involved in the infection-related liver damage.

Excess reactive oxygen species (ROS)-caused oxidative stress acts as a crucial mediator in acute liver injury (Prieto and Monsalve, 2017). The accumulation of ROS following hypoxia and shock leads to hepatocellular toxicity and liver damage (Macdonald et al., 2003). As an organ particularly susceptible to oxidative stress, the liver is equipped with the antioxidant enzymes to scavenge ROS, such as superoxide dismutases (SODs). SODs are the special cellar antioxidant enzymes for their responsible role of eliminating superoxide ion in the process of defensing against oxidative stress. Studies suggested that enhanced expression or activity of SODs attempted to minimize the liver injury (Cichoż-Lach and Michalak, 2014; El-Shabrawi et al., 2014). In this study, we observed that the expression of ERRγ was significantly reduced in the liver upon LPS challenge accompanied by the down-regulation of SOD1, SOD2 and SOD3, and meanwhile, DY131 upregulated both the expression of ERRγ (data not shown) and the SODs. Consistently, the production of hepatocytic ROS induced by LPS was attenuated by DY131 treatment. These data suggested that DY131 had a possible role in increasing the expression of SODs and the clearance of ROS.

In septic liver injury, excessive inflammatory mediators are produced in both hepatocytes and other cells, leading to a local and systemic inflammation (Siore et al., 2005). The persistent release of cytokines, such as TNF-α and IL-6, induces hepatocellular inflammation and apoptosis, ultimately leading to liver injury (Jiang et al., 2018). The inhibition of inflammatory cytokines serves as a potential interventional strategy in the treatment of sepsis-induced liver injury (Jiang et al., 2018; Xiong et al., 2017). The function of ERRγ in inflammatory diseases has not been clearly understood (Ambhore et al., 2018; Son and Chun, 2018; Yuk et al., 2015). Kim et al. suggested that ERRγ was a negative regulator of osteoclastogenesis and protected against inflammatory bone loss (Kim H.-J. et al., 2019). Son et al. found that ERRγ was upregulated in cartilage from patients with osteoarthritis and contributed to cartilage destructions in mice (Son et al., 2017). Similarly, other subtypes of ERRs had protective or detrimental roles in inflammatory responses (Sartoretto et al., 2019; Yuk et al., 2015). In this study, both circulatory and liver inflammatory cytokines were significantly reduced in LPS-challenged mice pretreated with ERRγ agonist DY131. In agreement with these observations, results of histological analyses and TUNEL staining further confirmed the *in vivo* protective effect of ERRγ agonist against LPS-induced liver injury.

According to the transcriptomics data, we found that LPS treatment significantly upregulated the inflammation-related genes, chemokines and the genes involved in apoptosis in liver, while most of them were suppressed by DY131 administration. KEGG analysis has identified the TNF signaling pathway as the top one significantly enriched pathway from DEGs that were upregulated in LPS-treated group while suppressed in the DY131-treated group. TNF-α is the key inflammatory factor involved in the progression of sepsis (Ertel et al., 1994; Georgescu et al., 2020; Parameswaran and Patial, 2010; Spinas et al., 1992). In clinical research, TNF-α was considered as the initial factor that triggered and mediated the sepsis (Parameswaran and Patial, 2010). TNF-α level was dramatically increased in the serum from patients with multiple causes-induced septic shock, and was positively correlated with disease severity and prognosis (Georgescu et al., 2020). Thus, TNF-α and its related signaling pathways are important targets for the treatment of sepsis. Studies have highlighted a role of TNF-α in regulating the expression of interferon regulatory factor 1 (IRF1), C-X-C motif chemokine ligand 2 (CXCL2) and colony stimulating factor 2 (CSF3) in inflammatory disease models (Bonelli et al., 2019; Slowikowski et al., 2020; Koeffler et al., 1987), and these inflammatory mediators were all filtered by RNA-Seq. Our results demonstrated an effective role of DY131 on anti-TNF-α signaling, indicating that DY131 might improve acute liver injury through suppressing this pathway to some extent. On the other hand, DEGs that were downregulated after LPS treatment and then rescued by DY131 treatment enriched the metabolic pathway as the most significantly changed pathway. Recent reports indicated that ERRγ played an important role in the regulation of metabolic events and that it was in charge of many physiological and pathological processes (Misra et al., 2017). DY131 is an agonist that can induce the transcriptional activity of ERRγ and regulate ERRγ-mediated metabolism (Huang et al., 2020; Yu and Forman, 2005). Studies have demonstrated that the altered and disrupted metabolic homeostasis served as the crucial factor in sepsis, including LPS-induced acute liver injury (Liao et al., 2016). These studies concluded that LPS could severely impair several important liver metabolic pathways, such as tricarboxylic acid (TCA) and urea cycles, gluconeogenesis, glycolysis and so on (Feng et al., 2013; Geng et al., 2020). In our study, genes regarding hepatic metabolism were also investigated and analyzed. For instance, liver glutathione S-transferases (GSTAs) and phenylalanine hydroxylase (PAH) were deficient upon LPS challenge and could be partially restored by DY131 (Pham et al., 2004; Richards et al., 2020). Succinyl-CoA ligase (SUCLG1), an important enzyme in TCA cycle, was also reversed by DY131 to a certain degree (Donti et al., 2016). The change of carbamoyl phosphate synthetase-1 (CPS1), a major enzyme involved in urea cycle, had a similar pattern as revealed by RNA-Seq (Park et al., 2019). These data suggested that DY131 could improve the disrupted metabolism during LPS-induced liver injury. However, further studies are needed to clarify the detailed mechanism.

In summary, the present study demonstrated that ERRγ agonist DY131 could attenuate oxidative stress, inflammation and apoptosis, and overall protect against LPS-induced acute liver injury. These findings provide evidence that targeting ERRγ might be a potential therapeutic strategy in the treatment of sepsis-associated liver injury.

## Data Availability

We have uploaded the RAW data of RNA sequencing to the public repository Gene Expression Omnibus and the review link as below: https://www.ncbi.nlm.nih.gov/geo/query/acc.cgi?acc=GSE16088.
