# Supplementary figures and images for: Estrogen-Related Receptor γ Agonist DY131 Ameliorates Lipopolysaccharide-Induced Acute Liver Injury
Source: Front Pharmacol. 2021 Apr 23;12:626166. doi: 10.3389/fphar.2021.626166 (PMC8104008; doi:10.3389/fphar.2021.626166)

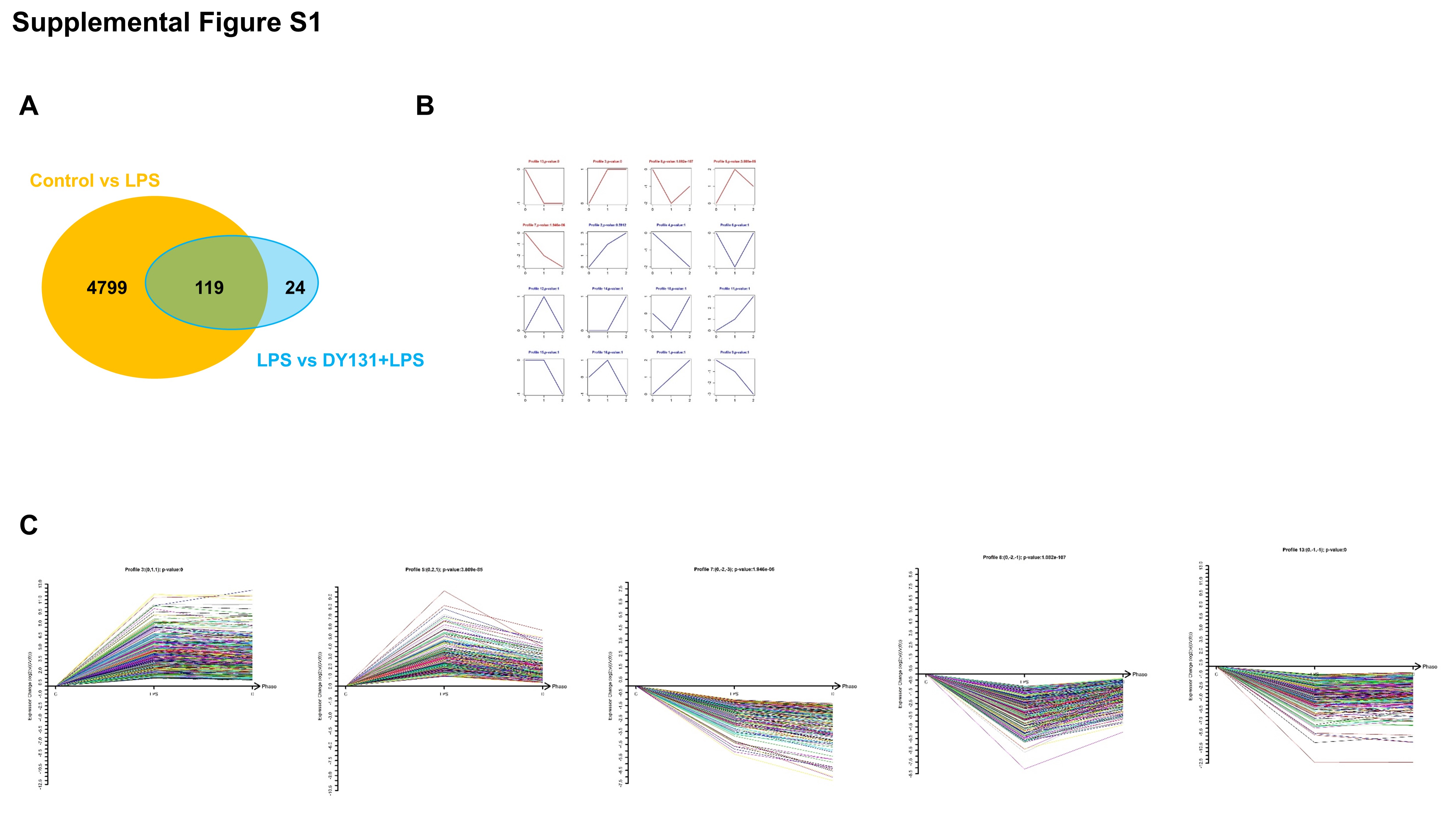

Supplement: Supplementary file 1 [file image1.jpeg]
