# Supplementary material for: Estrogen-Related Receptor γ Agonist DY131 Ameliorates Lipopolysaccharide-Induced Acute Liver Injury
Source: Front Pharmacol. 2021 Apr 23;12:626166. doi: 10.3389/fphar.2021.626166 (PMC8104008; doi:10.3389/fphar.2021.626166)
Supplement: Supplementary file 2 [file datasheet1.pdf]

Fig 1 B

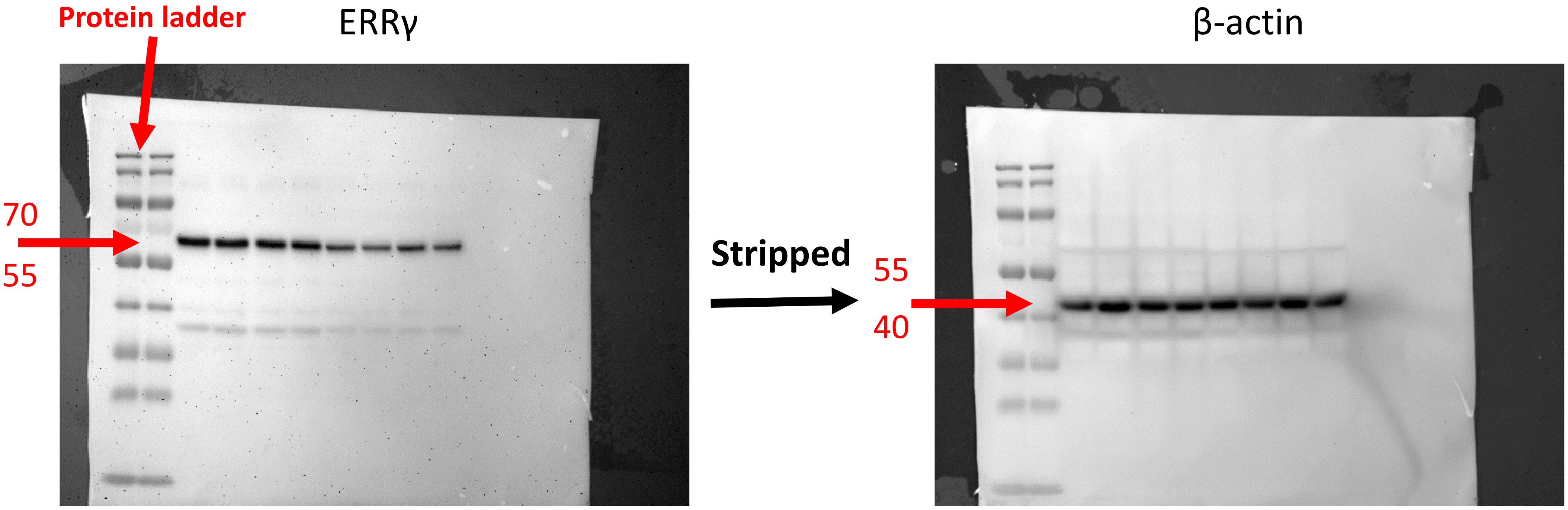

7.5% PAGE Gel

PVDF1 Sample order: protein ladder1/2, control 1/2/3/4, LPS 1/2/3/4

Products: PageRuler prestained protein ladder (thermo scientific 26616)
Stripping buffer (beyotime P0025)

Fig 1 B

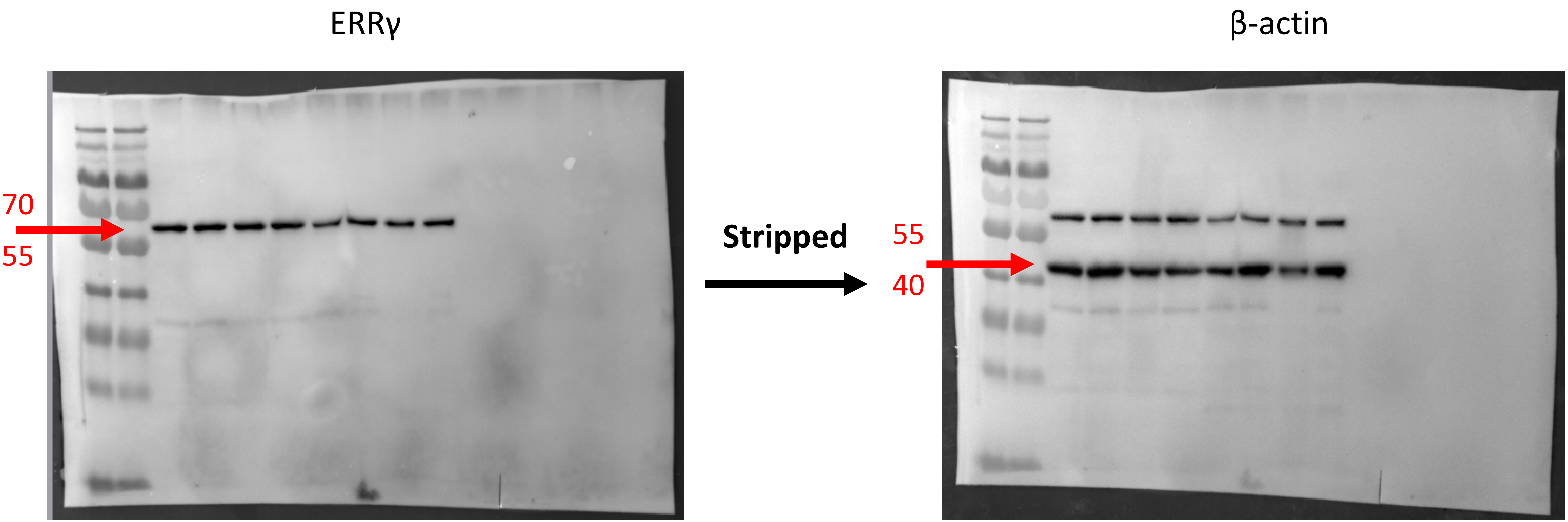

7.5% PAGE Gel

PVDF2 Sample order: protein ladder 1/2, control 5/6/7/8, LPS 5/6/7/8

Fig 3 D

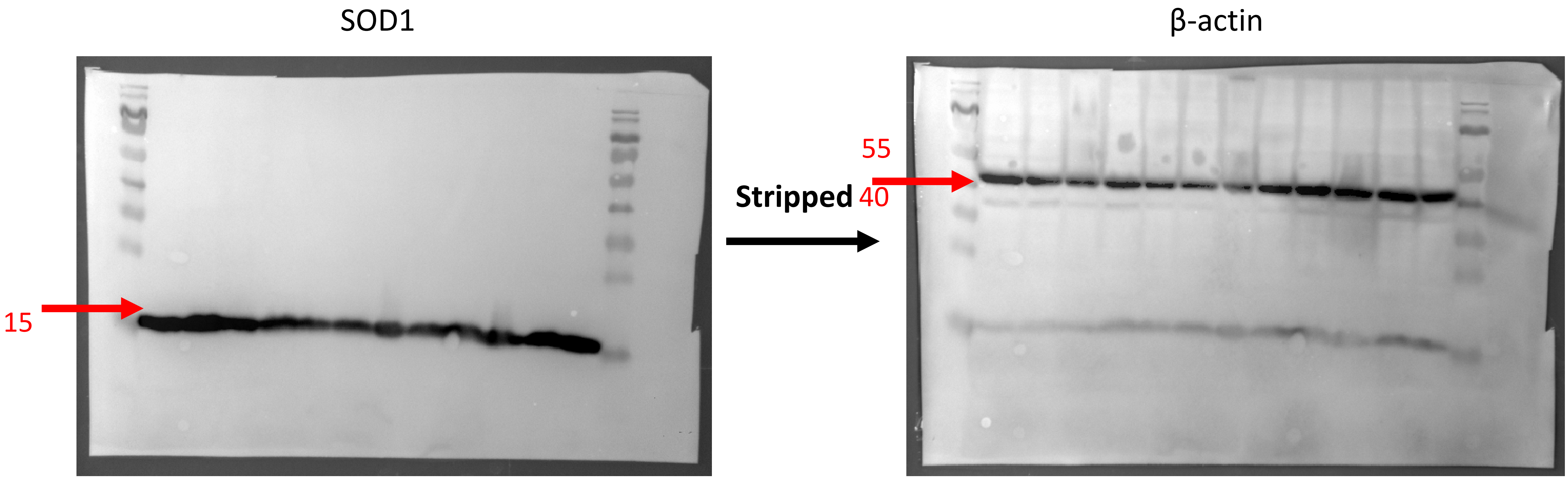

12.5% PAGE Gel

PVDF1 Sample order: protein ladder, control 1/2/3/4, LPS 1/2/3/4, DY131+LPS 1/2/3/4

Fig 3 D

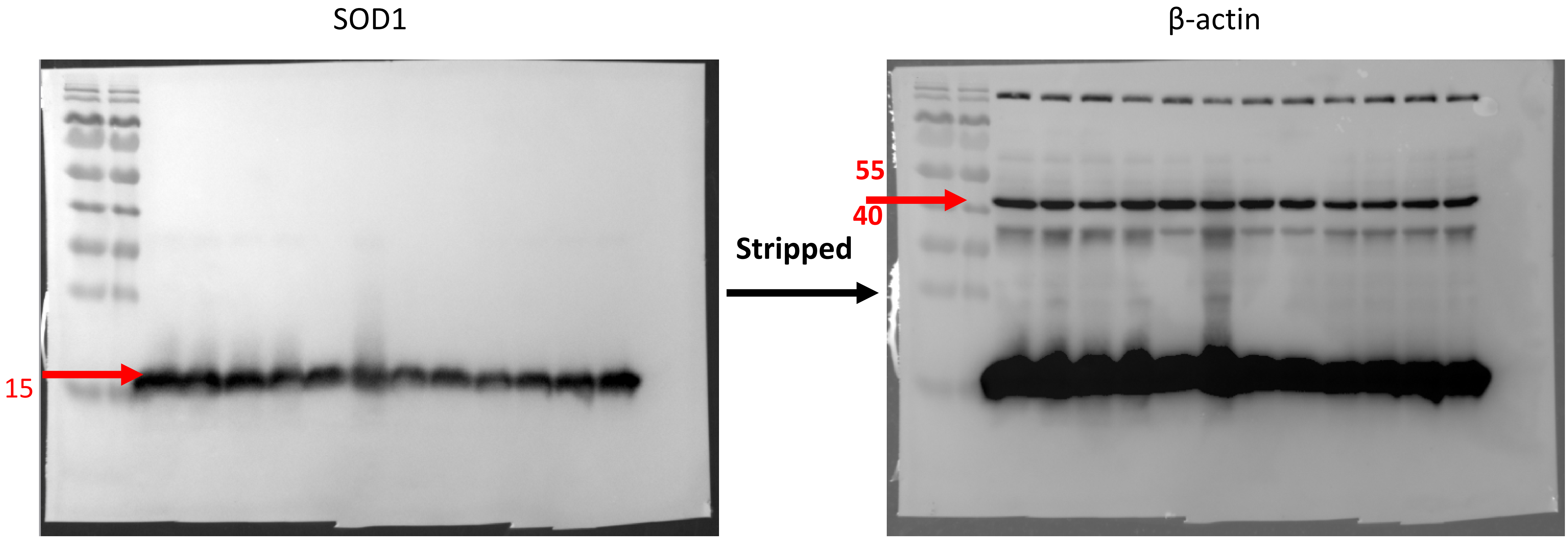

12.5% PAGE Gel

PVDF2 Sample order: protein ladder 1/2, control 5/6/7/8, LPS 5/6/7/8, DY131+LPS 5/6/7/8

Fig 3 D

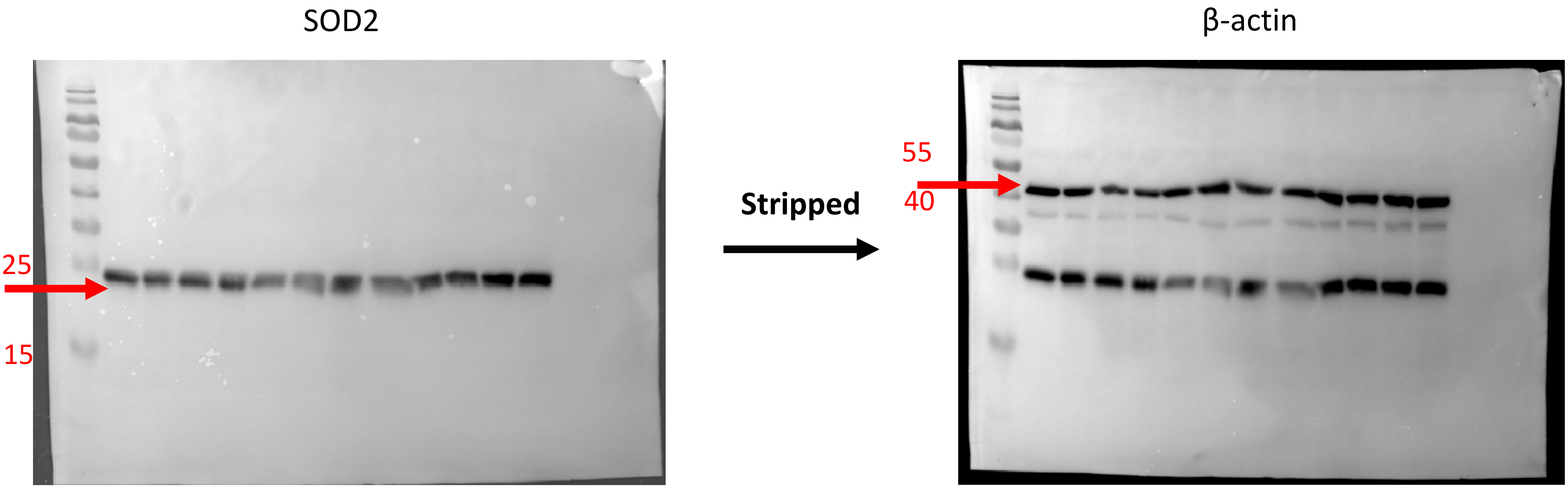

12.5% PAGE Gel

PVDF1 Sample order: protein ladder, control 1/2/3/4, LPS 1/2/3/4, DY131+LPS 1/2/3/4

Fig 3 D

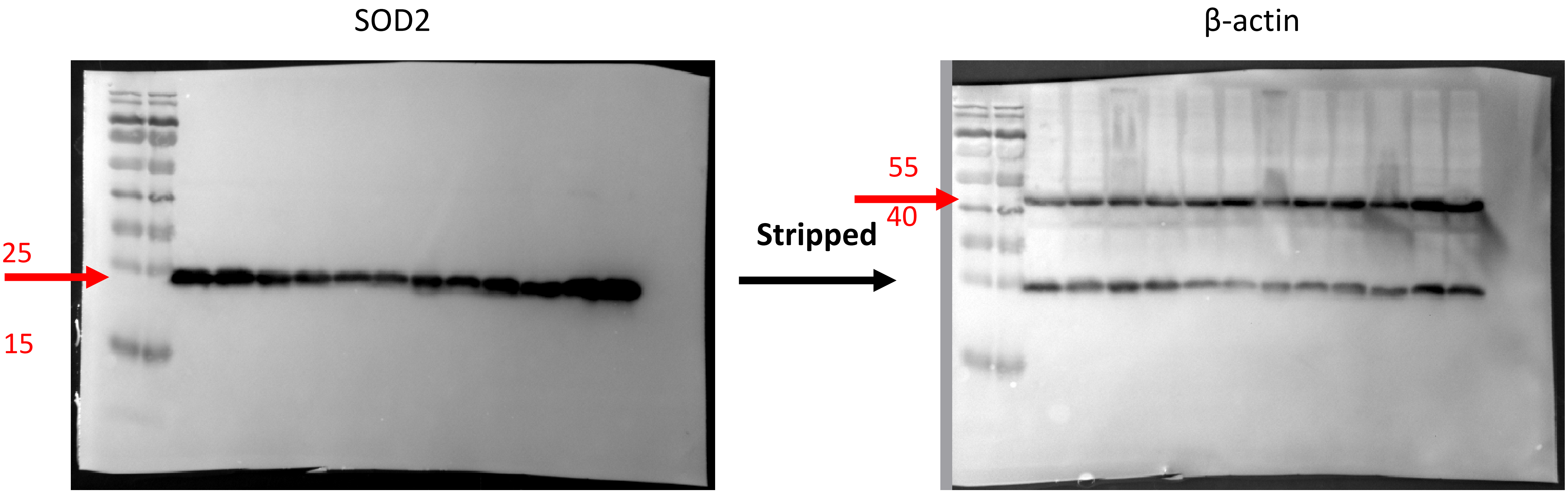

12.5% PAGE Gel

PVDF2 Sample order: protein ladder 1/2, control 5/6/7/8, LPS 5/6/7/8, DY131+LPS 5/6/7/8

Fig 3 D

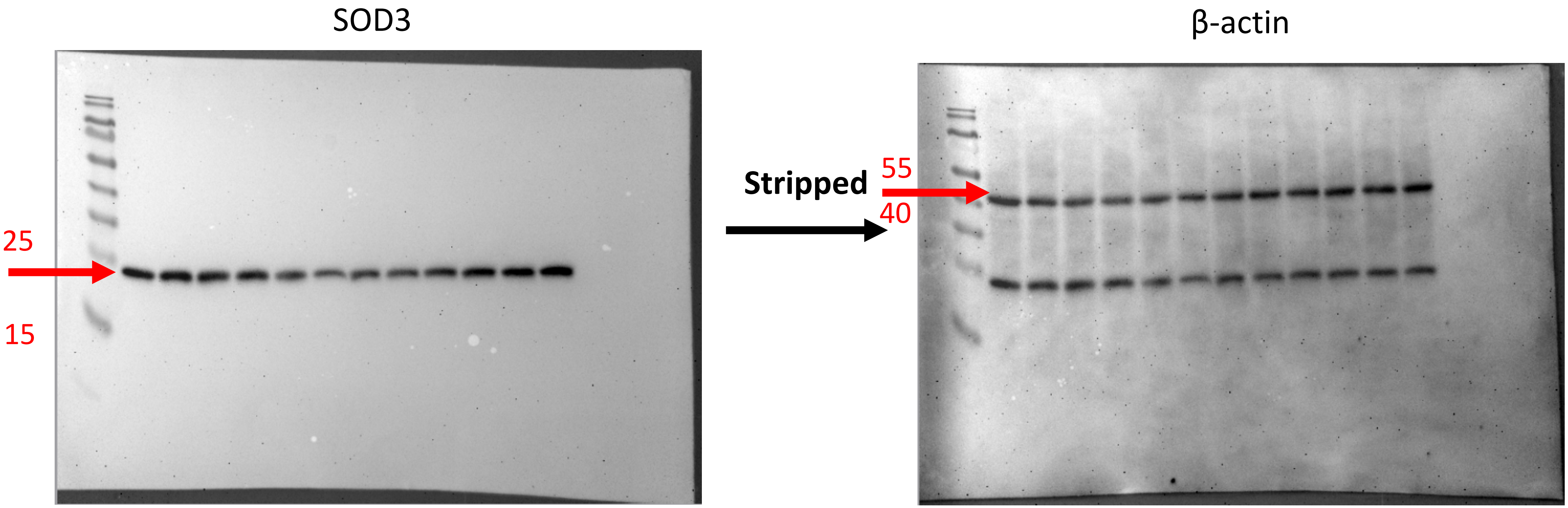

12.5% PAGE Gel

PVDF1 Sample order: protein ladder, control 1/2/3/4, LPS 1/2/3/4, DY131+LPS 1/2/3/4

Fig 3 D

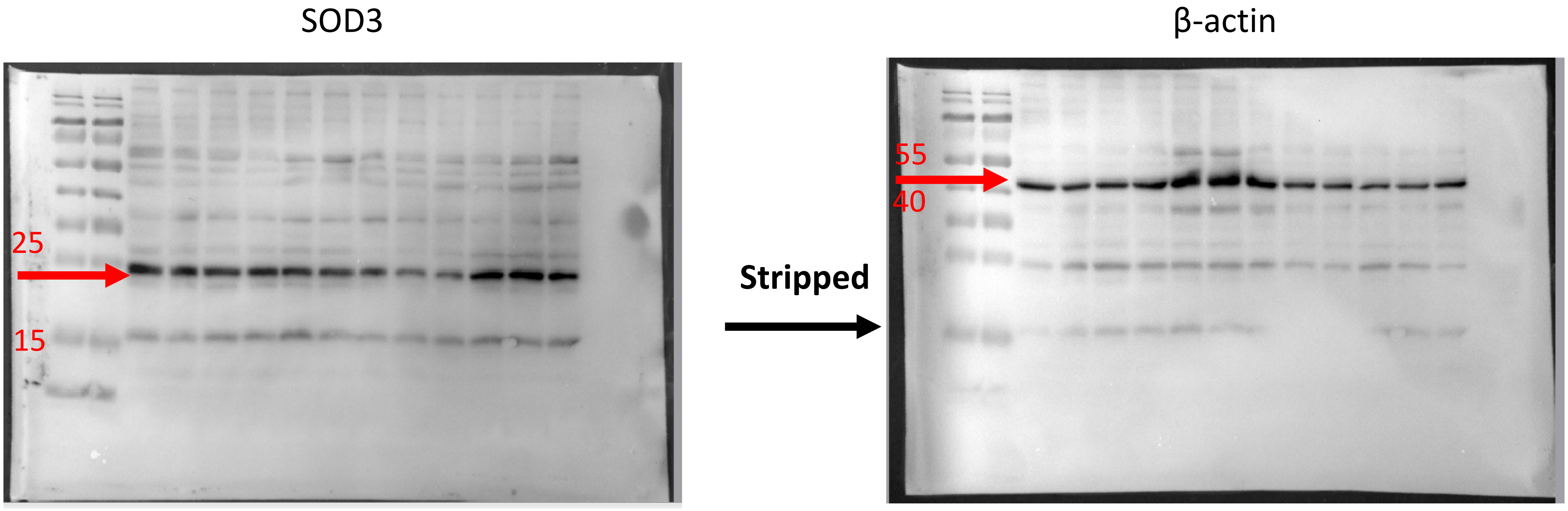

12.5% PAGE Gel

PVDF2 Sample order: protein ladder 1/2, control 5/6/7/8, LPS 5/6/7/8, DY131+LPS 5/6/7/8

Fig 5 B

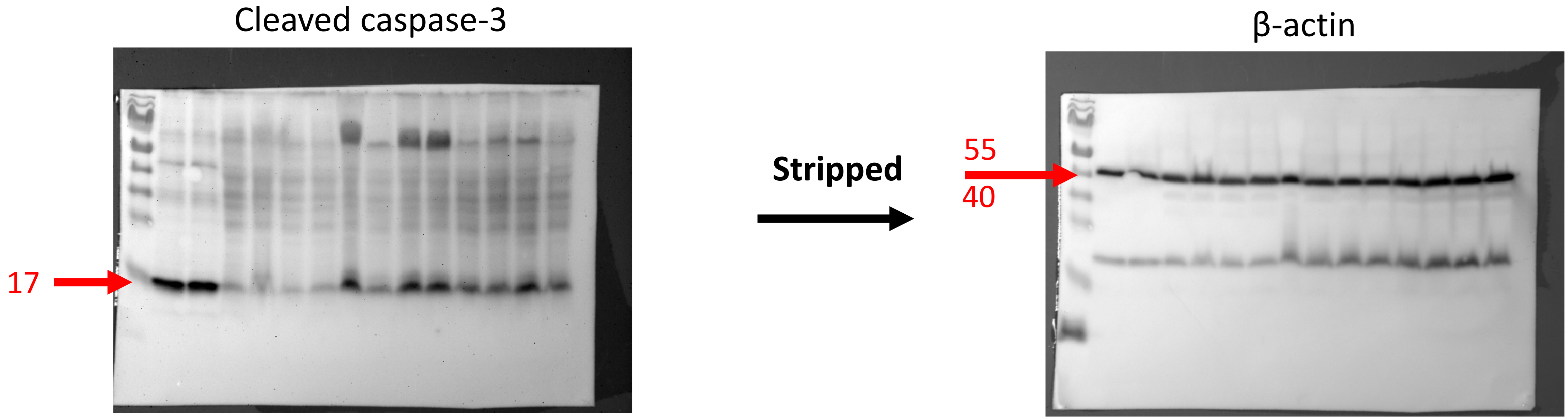

12.5% PAGE Gel

PVDF1 Sample order: protein ladder,
positive control 1/2,
control 1/2/3/4,
LPS 1/2/3/4,
DY131+LPS 1/2/3/4

Positive ctrl: cells treated with TNF cytokine (20ng/ml)

Fig 5 B

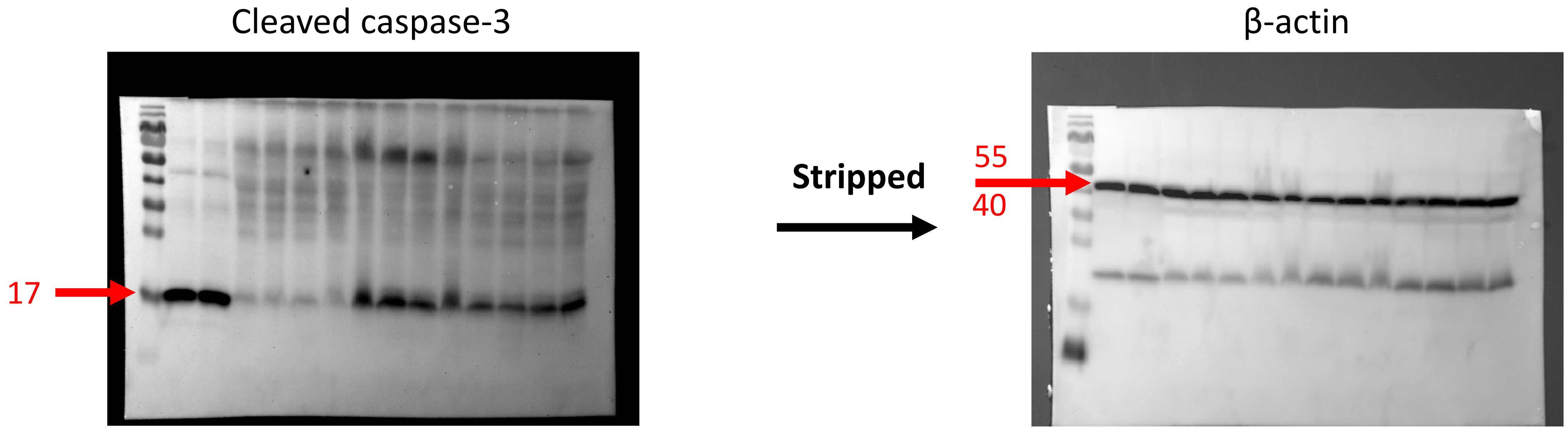

12.5% PAGE Gel

PVDF2 Sample order: protein ladder,
positive control 1/2,
control 5/6/7/8,
LPS 5/6/7/8,
DY131+LPS 5/6/7/8

Positive ctrl: cells treated with TNF cytokine (20ng/ml)

Fig 5 B

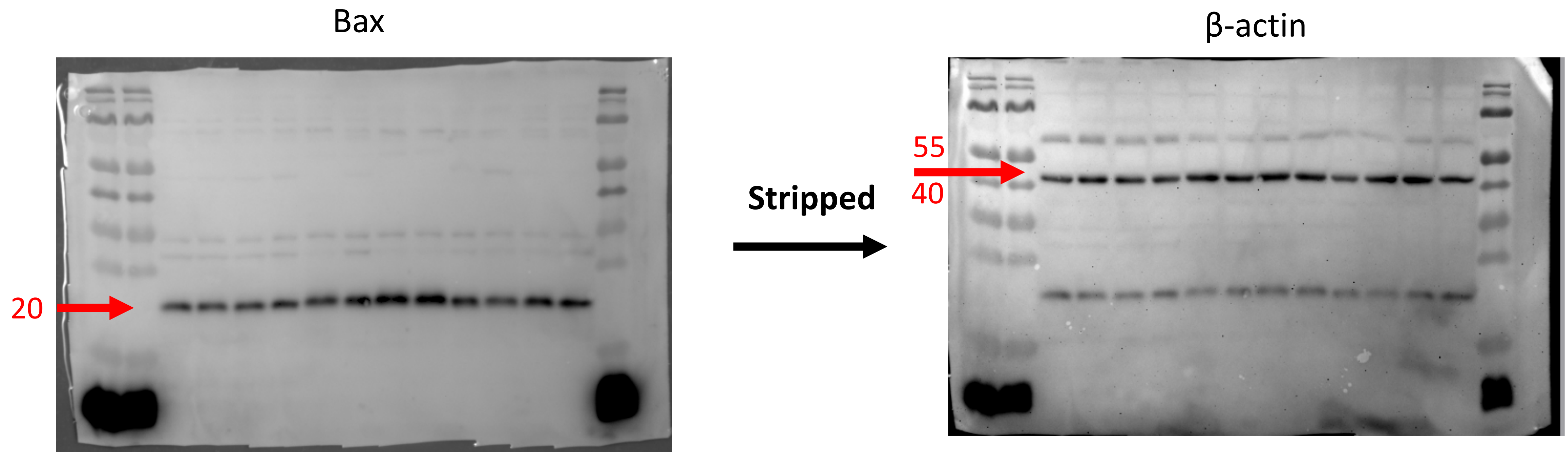

12.5% PAGE Gel

PVDF1 Sample order: protein ladder, control 1/2/3/4, LPS 1/2/3/4, DY131+LPS 1/2/3/4

Fig 5 B

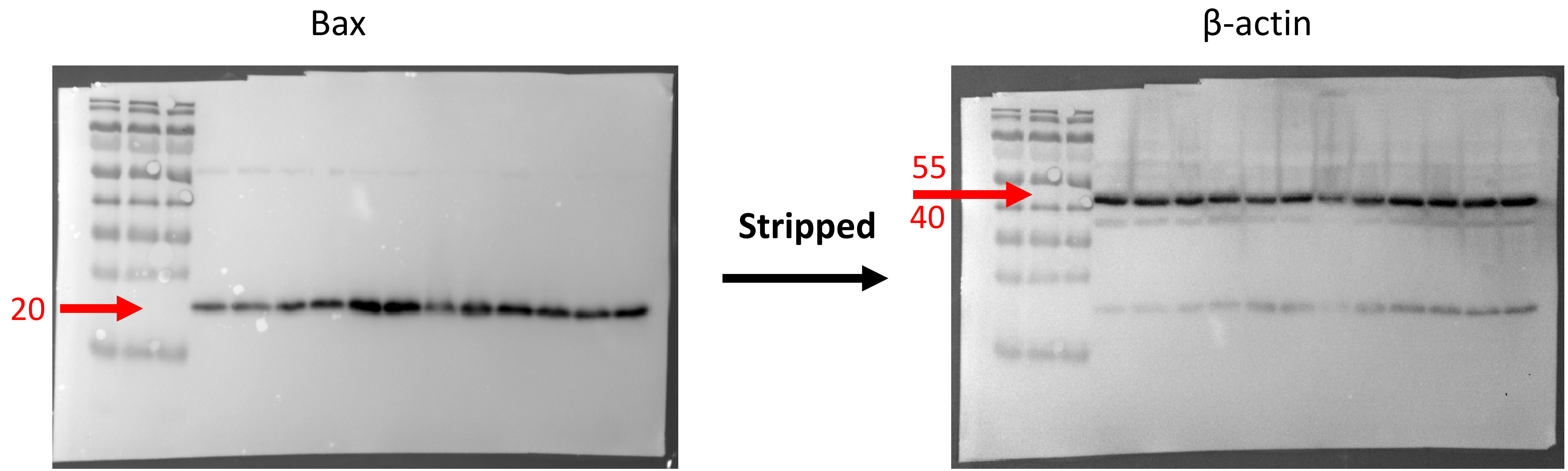

12.5% PAGE Gel

PVDF2 Sample order: protein ladder, control 5/6/7/8, LPS 5/6/7/8, DY131+LPS 5/6/7/8
